# Folic Acid–Functionalized Metal-Organic Framework Nanoparticles as Drug Carriers Improved Bufalin Antitumor Activity Against Breast Cancer

**DOI:** 10.3389/fphar.2021.747992

**Published:** 2022-01-18

**Authors:** Hairong Zeng, Chao Xia, Bei Zhao, Mengmeng Zhu, HaoYue Zhang, Die Zhang, Xin Rui, Huili Li, Yi Yuan

**Affiliations:** ^1^ Department of Pharmacy, Putuo Hospital, Shanghai University of Traditional Chinese Medicine, Shanghai, China; ^2^ Institute of Interdisciplinary Integrative Medicine Research, Shanghai University of Traditional Chinese Medicine, Shanghai, China; ^3^ Interventional Cancer Institute of Chinese Integrative Medicine, Shanghai University of Traditional Chinese Medicine, Shanghai, China; ^4^ Engineering Research Center for Nanophotonics and Advanced Instrument, Ministry of Education, School of Physics and Electronic Science, East China Normal University, Shanghai, China; ^5^ Colorectal Disease Center of Nanjing Hospital of Chinese Medicine Affiliated to Nanjing University of Chinese Medicine, Nanjing, China; ^6^ Baoshan Branch, Shuguang Hospital Affiliated to Shanghai University of Traditional Chinese Medicine, Shanghai, China

**Keywords:** bufalin, MOF, breast cancer, drug delivery, antitumor, tumor targeting

## Abstract

Bufalin (Buf), an active ingredient of the traditional Chinese medicine Chansu, is known to have anticancer effects for breast cancer. However, its poor solubility, high toxicity, and extensive side effects limit its use. Metal-organic frameworks (MOFs) are a class of promising drug delivery systems known for their high porosity. Here, we designed and constructed pH-sensitive and redox-responsive folic acid–modified MOFs as drug carriers of Buf (FA-MOF/Buf). Moreover, the anticancer activity of nanomedicines was also explored *in vitro* and *in vivo*. Compared to free Buf, the FA-MOF/Buf nanoparticles demonstrated improved water solubility and stability, higher intracellular uptake, and enhanced cytotoxicity in breast cancer cells *in vitro*. Furthermore, it displayed improved accumulation in the tumor site, enhanced anticancer activity, and reduced side effects *in vivo*. Our results demonstrated that FA-MOF could be developed as a potential delivery system for Buf to improve its antitumor activity for breast cancer treatment.

## Introduction

Bufalin (Buf) is one of the active ingredients of Chansu, which has been used for cancer therapy in traditional Chinese medicine for nearly a thousand years (Xu et al., 2019). Recently, studies confirmed that Buf has a significant antitumor effect against various cancers, including breast cancer (Li et al., 2018), liver cancer (Miao et al., 2013), gastric cancer (Zhao et al., 2017), lung cancer (Kang et al., 2017), and colorectal cancer (Zhang et al., 2016). However, the clinical utility of Buf is limited due to its cardiotoxicity, insolubility in water, short half-life, and fast metabolism (Bick et al., 2002). Thus, exploring novel therapeutic strategies to decrease its side effects and enhance its bioavailability has significant clinical importance. Gao et al. designed and optimized a PEGylated BF211 liposome (BF211@Lipo), which showed prolonged blood circulation time, reduced cardiotoxicity, and improved antitumor activity (Gao et al., 2021). Xu et al. synthesized a bufalin-loaded nanosphere: CaP/1,2-bis(diphenylphosphino) ethane-polyethylene glycol (CaP/DPPE-PEG) with covalently attached epidermal growth factor (CaP/DPPE-PEG-EGF NSs) used as carriers for the delivery of bufalin for targeted antitumor therapy, which can increase bufalin’s stability and absorption rate and improve its efficacy (Xu et al., 2019). Hu et al. prepared a bufalin-loaded pluronic PEI nanoparticle, which showed a significantly enhanced potential of nanoparticles to deliver anticancer drugs, to enhance antitumor effects (Hu et al., 2014). Chen et al. prepared a transferrin (Tf) and folic acid (FA) co-modified bufalin (BF) liposome, which showed significantly suppressed tumor growth (Chen and Liu, 2018). Although lots of nanoparticle delivery systems have been used to improve the efficacy of bufalin in antitumor therapy, the stability, drug loading, targeting, and safety issues still limit its use, urging for new delivery systems to solve these problems.

Metal-organic frameworks (MOFs) are a class of hybrid porous materials which was assembled based on the coordination effect between metal ions and function groups of organic ligands (Wu and Yang, 2017). MOFs have demonstrated advantageous characteristics as biomaterials, such as high pore volume, large internal surface area, uniform porosity, and high stability (Zhang H. et al., 2017). Due to the abovementioned superior properties of MOFs, they are widely used in biomedical applications, such as enzyme catalysis (Mehta et al., 2016), gas storage (Millward and Yaghi, 2005), pH sensing (He L. et al., 2014), diagnosis (Shi et al., 2018), photovoltaic applications (Luo et al., 2019), and drug delivery (Chen et al., 2018; Sun et al., 2020). MOFs synthesized using various metals are used as drug delivery systems, such as Cr, Fe, Zn, and Zr. Férey et al. have reported that a Cr-based MOF (MIL-100) exhibited remarkable IBU adsorption (Horcajada et al., 2006a). Horcajada et al. reported an Fe-based MOF (MIL-53) as a drug delivery system (Horcajada et al., 2008b). Chen et al. reported a Zn-based MOF load of glucose oxidase (GOx) (ZIF8) as versatile glucose-responsive nanocomposite that may act as autonomous, sense-and-treat vehicles for controlling diabetes or macular diseases (Chen WH. et al., 2018; Chen W.-H. et al., 2018). Recently, taking into account the toxicity of chromium compounds, some low-toxicity metals have been applied for the MOFs. For example, Zr-based MOFs have received increasing attention based on nontoxicities, excellent stabilities, and biocompatibility (Vermoortele et al., 2013). He et al. reported a Zr-based MOF for the delivery of cisplatin, enhancing their therapeutic efficacy by overcoming drug resistance in ovarian cancer cells (He C. et al., 2014). Fu et al. synthesized new-style flexible Mn-doped zirconium metal-organic framework (Mn-ZrMOF) nanocubes, which were demonstrated to be an effective microwave-sensitive agent and efficiently suppressed the tumor cell growth (Fu et al., 2018). Zhou et al. reported triphenyl phosphate (TPP)–conjugated and doxorubicin (DOX)-loaded porous zirconium metal-organic framework nanocubes (ZrMOF NCs) modified by polyethylene glycol (PEG) and ZrMOF-PEG-TPP@DOX NCs as the microwave sensitizer with mitochondria target enhancing microwave thermal therapy against tumors (Zhou et al., 2018). Li et al. synthesized a kind of open-mouthed Zr MOF-derived nano-popcorns (ZDNPs), having a higher microwave–thermo conversion efficiency than UIO-66 (Li et al., 2020).

Moreover, the organic components of MOFs that reach the reactive groups or active sites, such as-COO-, CH3COO-, or -OH, are accessible for post-synthetic modifications (PSMs) through covalent attachment of other organic molecules, allowing formulation of new chemical functionalities (Zhang F. et al., 2017). In recent decades, various MOFs and MOFs with PSMs were used as drug carriers for disease therapy, particularly for cancer treatment. Pang et al. synthesized a bone-targeting immunostimulatory MOF (BTisMOF) nanoparticle (functionalized immunostimulatory cytosine−phosphate−guanosine (CpG)–loaded MOF nanoparticles with bone-targeting capabilities by surface modification with FDA-approved antiresorptive bisphosphonate, zoledronic acid) for the treatment of breast cancer bone metastases (Pang et al., 2020). Compared with other drug carriers and nanoparticles (NPs), MOF NPs possess the advantages of having adjustable structures, large pore sizes for drug loading, and biodegradability (Cai et al., 2017). Additionally, pH-sensitive and redox-responsive MOFs were also designed to utilize the microenvironment of tumor sites, such as low pH and high glutathione (GSH) concentration (Chen et al., 2016; Ang et al., 2017). Moreover, attachment of folic acid (FA) to these drug carriers can be an effective strategy for active targeting in drug delivery for cancer cells (Shi et al., 2018) because folate receptors (FRs) are overexpressed on the cancer cell surface (Wong et al., 2015).

**SCHEME 1:**
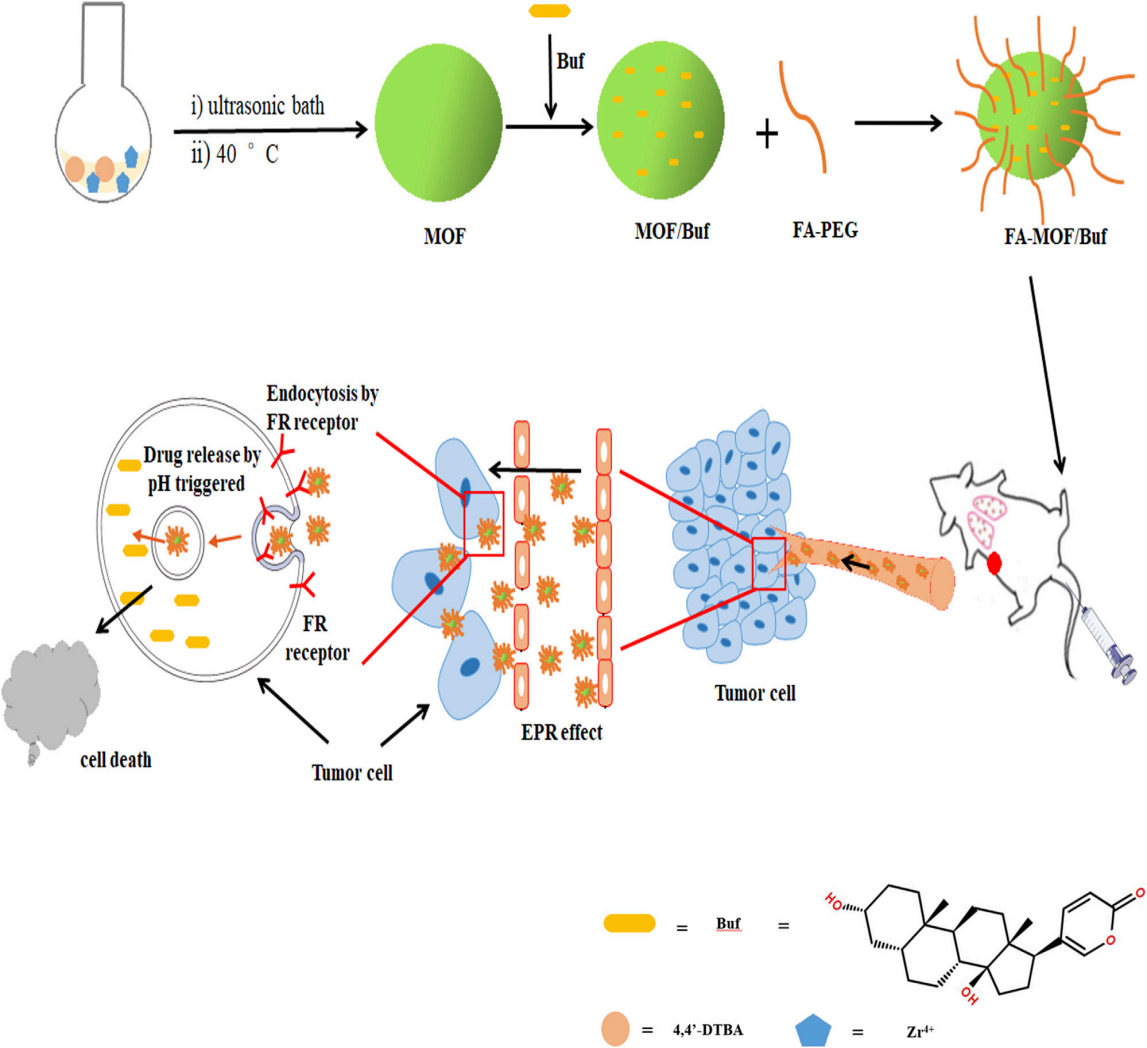
Schematic illustration of the preparation of FA-MOF/Buf nanoparticles and the release of active drug (Bufalin, Buf) to tumor sites.

In this study, we used the GSH-sensitive organic ligands 4,4-dithiobisbenzoic acid (4,4′-DTBA) and ZrCl_4_ to design a pH-sensitive and redox-responsive MOF drug carrier. We then incorporated Buf into MOFs and FA onto the MOF surface. The obtained FA-MOF/Buf NPs remain firm and stable at neutral conditions and quickly degrade in a low pH tumor environment of approximately 6.5–6.8 (Chen et al., 2018; Chen et al., 2019). The FRs on cancer cell surfaces can identify FA on the NPs, promoting selective uptake through receptor-mediated endocytosis and the enhanced permeability and retention (EPR) effect (Chen et al., 2018; Narmani et al., 2019). Once the loaded carrier system enters the cancer cells, the disulfide bonds in the MOFs are cleaved by GSH, whose intracellular concentration is 2–10 mM in cancer cells and 2–20 μM in normal cells (Albini and Sporn, 2007; Lei et al., 2018). This cleavage triggers the disassembly of the MOF and release of free Buf. Through this method, the low solubility and poor stability of Buf are addressed, and the tumor cells can be rapidly eliminated with the high intracellular concentration of free drugs (Scheme 1). Our results suggest that FA-MOF/Buf NPs can increase the curative effect of Buf in the treatment of breast cancer.

## Materials and Methods

### Materials

Buf ( ≥ 99% purity) was purchased from Chengdu Puruifa Technology Development Co., Ltd. (Chengdu, China). Cyanine 5 amine (Cy5) was purchased from Lumiprobe (Hallandale Beach, FL, United States). Coumarin-6 (C6) was purchased from Sigma-Aldrich Corporation (St. Louis, MO, United States). 4,6-Diamidino-2-phenylindole (DAPI) was obtained from Beyotime Biotech (Shanghai, China), while 4,4′-DTBA was purchased from Absin Bioscience Inc. (Shanghai, China). ZrCl4 was purchased from Aladdin (Shanghai, China). FA-PEG (MW = 2000 Da) was obtained from Shanghai Pengsheng Biotechnology Co., Ltd (Shanghai, China). CCK-8 was purchased from Dojindo (Kumamoto, Japan). Dulbecco’s modified Eagle’s medium (DMEM) and phosphate-buffered saline (PBS) were obtained from Hyclone (Thermo Fisher Scientific, Waltham, MA, United States). Trypsin/EDTA solution, penicillin, streptomycin, and FBS were obtained from Gibco BRL (Carlsbad, CA, United States).

### Synthesis and Characterization of FA-MOF/Buf NPs

For the synthesis of MOFs (MOF-Zr) (Lei et al., 2018), 1.2 mmol ZrCl4 and 4,4′-DTBA were added into a Teflon-capped glass flask containing 30 ml of dimethylformamide (DMF) and 1 ml of acetate. The mixture was nested and placed in an ultrasonic bath for 5 min at room temperature until the solution was clear. Then, the mixture was incubated at 40°C until it became milky. Finally, the resulting powder was obtained through centrifugation at 8,000 × g for 10 min, washed three times with DMF and ethanol, and then dried at 35°C under vacuum. To obtain the MOF/Buf, 10 mg of the MOF was added into 5 ml of methanol containing Buf (0.4 mg/ml) and was stirred for 24 h under dark conditions. The MOF/Buf was obtained by centrifugation, washed three times with methanol, and dried at 35°C under vacuum. For the preparation of FA-MOF/Buf, FA-PEG was adsorbed on the surface of MOF/Buf through a mechanical mixing method (Horcajada et al., 2010; Shi et al., 2018). In brief, FA-PEG was dissolved in deionized water at a 10-mg/ml concentration and then MOF/Buf was added to this solution. Afterward, the mixture was sonicated for 10 min and stirred at room temperature for 48 h. The obtained FA-MOF/Buf product was collected by centrifugation and was dried at 35 °C under vacuum. The NP size distribution and zeta potential of the samples were measured by dynamic light scattering (DLS) (ZEN3600, Malvern Instruments Ltd., Malvern, Worcestershire, United Kingdom). The shape and size of the samples were identified using scanning electron microscopy (S-4800, Hitachi, Tokyo, Japan) and transmission electron microscopy (TEM) (JEM-1200EX, JEOL, Tokyo, Japan). The functional group modifications of the particles were confirmed by Fourier Transform Infrared Spectroscopy (FT-IR) (IR-460, Shimadzu, Kyoto, Japan). The gas adsorption/desorption isotherms for N_2_ at 77 K were performed on a Tristar 3000 analyzer. The data of Raman spectrum were collected by the Raman imaging microscope (DXR3xi, Thermo Fisher Scientific, United States).

### Drug Loading Efficacy of FA-MOF/Buf NPs

The measurement of the Buf loading amount was obtained at 298 nm by UV–Vis spectroscopy. The standard curve between the absorbance and Buf concentration was obtained. The Buf concentration in the acid solution was calculated according to the standard Buf concentration curve. In the acid solution, 20 mg FA-MOF/Buf NPs were dissolved in 1 mol/L HCl and diluted by deionized water containing 10 mmol/L GSH and 1% Tween 80. The Buf drug loading efficiency (LE%) on NPs was calculated using the following equation:

LE(%)=(Weight of Buf loaded)(Weight of FA−MOF/Buf NPs)×100%, (1)

### Release of Buf From FA-MOF/Buf NPs

Release assays were carried out in PBS at 37°C. To avoid the interference from the carrier, the release experiment was completed by the dynamic dialysis method using a dialysis membrane (MWCO: 1,000 Da). In brief, 20 mg FA-MOF/Buf NPs was placed into a 50-ml centrifuge tube with PBS solution (pH 5.5 + GSH 0 mM, pH 6.5+ GSH 0 mM and pH 7.4+ GSH 0 mM) or PBS solution (pH 6.5+ GSH 10 mM and pH 7.4+ GSH 10 mM). The release experiments were then performed at 37°C with shaking (100 rpm). Then, 1 ml of the sample was withdrawn at predetermined time intervals, and the amount of Buf released from FA-MOF/Buf NPs was analyzed. The sample was returned to the original solution to maintain a constant volume. The concentration of Buf in the samples was detected by UV–Vis spectroscopy at 298 nm. The cumulative release percentage of Buf was calculated as follows:

CR(%)=MrML×100%, (2)

where Mr and ML are the amounts of released drug and loaded drug, respectively.

### Cell Culture

The breast cancer cells MDA-MB-231, MCF-7, and 4T1 were purchased from the Cell Bank of the Chinese Academy of Sciences (Shanghai, China). Both cells were cultured in folic acid–free DMEM containing 10% fetal bovine serum (FBS), 100 U/mL penicillin, and 100 μg/ml streptomycin at 37°C in a humidified atmosphere containing 5% CO_2_.

### *In vitro* Cytotoxicity

The cytotoxicity of free Buf, MOF/Buf, and FA-MOF/Buf against MDA-MB-231 and MCF-7 cells was assessed using the CCK-8 assay. In brief, cells were seeded into 96-well plates after overnight incubation at 37°C at a density of 1 × 10^4^ cells/well, and the medium was replaced with a fresh medium containing free Buf, MOF/Buf, and FA-MOF/Buf for 48-h incubation; the concentrations of Buf were based on the same free Buf and its counterpart of loaded Buf into MOF. To support the specificity of the FA-MOF/Buf constructs, a control of cytotoxic evaluation of MOF/Buf and FA-MOF/Buf on A549 cells (folate receptor-negative A549 lung carcinoma cells) was carried out (Chen et al., 2017). Finally, each well was added with 10 µL of CCK-8, and cells were further incubated for 2 h. The absorbance was determined by using a microplate reader (Thermo Fisher Scientific) at 450 nm.

### Apoptosis Analysis

The apoptosis induced by the FA-MOF/Buf NPs in MCF-7 cells was quantitated using the fluorescein isothiocyanate (FITC) annexin V/propidium iodide (PI) kit. The cells were seeded in six-well plates (2.5 × 10^5^ cells/well). After overnight incubation, the medium was replaced with a fresh medium containing free Buf, MOF/Buf, and FA-MOF/Buf, and the cells were further incubated for 48 h. Afterward, the cells were collected and stained with 5 µL FITC annexin V and 5 µL PI for 15 min in the dark. Finally, cell apoptosis was analyzed using FACS (BD Biosciences, San Jose, CA, United States).

### Cell Cycle Analysis

The cell cycle distribution of FA-MOF/Buf NPs in MCF-7 cells was quantitated using the cell cycle and apoptosis kit. Cells were seeded in six-well plates (2.5 × 10^5^ cells/well). After overnight incubation, the medium was replaced with a fresh medium containing free Buf, MOF/Buf, and FA-MOF/Buf, and the cells were incubated for another 48 h. The cells were collected and fixed with 70% ice-cold ethanol overnight. Then, the cells were stained with PI/RNase buffer for 30 min in the dark. Finally, the cell cycle distribution was analyzed using FACS (BD Biosciences).

### Cellular Uptake

The intracellular uptake of FA-MOF was assessed by loading the fluorescent dye C6 instead of Buf. The MCF-7 cells were seeded in a confocal dish at a density of 1 × 10^5^ cells/dish and cultured for 24 h. Then, the medium was replaced with a fresh folic acid–free medium containing free C6, C6-labeled MOF (MOF/C6), and C6-labeled FA-MOF (FA-MOF/C6), also, to perform folic acid competition assay, 1 mM folic acid combined with C6-labeled MOF (FA + MOF/C6) was added. Afterward, the cells were incubated for 2 h at 37°C. For measuring the experimental endpoint, the cells were washed with PBS three times, fixed with 4% paraformaldehyde, and stained with DAPI. Fluorescent images were observed using a confocal laser scanning microscope (CLSM). In addition, the cellular uptake efficiency was determined using FACS (Calibur; BD Biosciences).

### *In vivo* Biodistribution Examination

The tumor-targeting capability of FA-MOF/Buf was assessed by loading the fluorescent dye Cy5 instead of Buf. Cy5 was encapsulated in the MOF and FA-MOF. MOF/Cy5 and FA-MOF/Cy5 (200 μL, Cy5 concentration: 2.5 × 10^−5^ mol/L) were injected through the tail vein of 4T1 (folate receptor was overexpressed) (Chen et al., 2017; Pu et al., 2019) tumor–bearing xenografted mice. Fluorescence imaging of the tumor-bearing mice was performed at 0, 0.5, 4, 8, 24, and 48 h post injection using a small animal *in vivo* fluorescence imaging system (LIVIS Lumina series III (PerkinElmer, Waltham, MA, United States). After 48 h, the nude mice were euthanized. The tumors and main organs (heart, liver, spleen, lungs, and kidneys) were collected for examination of the *ex vivo* distribution using the same fluorescence imaging system.

### *In vivo* Antitumor Efficacy

Subcutaneous tumors were established in nude mice (BALB/c, female, 4–6 weeks old) by injecting 4T1 cells (folate receptor was overexpressed) (Chen et al., 2017; Pu et al., 2019) (2×10^6^ cells in 0.2 ml PBS) into the left shoulder of the nude mice. Once the tumor volumes reached 100 mm^3^, the mice were randomized into five groups with six mice per group: normal saline group, MOF group, Buf solution group (2 mg/kg Buf), MOF/Buf group (2 mg/kg Buf), and FA-MOF/Buf group (2 mg/kg Buf). Solutions were administered by intravenous injection through the tail vein every 2 days for 3 weeks. The tumor sizes and body weight were measured at regular intervals. Tumor sizes were measured using a caliper, and the tumor volume ((V) = a×b^2^/2 (where a represents the length of the tumor and b represents the width of the tumor)) was obtained. At the end of the treatment period, all mice were killed. The tumors and main organs (heart, liver, spleen, lungs, and kidneys) were harvested and fixed with 4% paraformaldehyde for histological analysis using hematoxylin–eosin (H&E) staining.

### Hematoxylin–Eosin and Immunohistochemistry Staining

Tissues slides of tumor and main organs (heart, liver, spleen, lungs, and kidneys) were stained with hematoxylin–eosin and observed by a microscope ( × 100 magnification) for five fields. The expression of Ki67 in tumor tissues was measured by immunohistochemistry; in brief, the slides were deparaffinized and rehydrated, then antigen retrieval and incubation with 3% H_2_O_2_ was performed for 25 min, followed by incubation with primary antibody of Ki67 (1:200) at 4°C overnight, and then incubated with secondary antibody at 37°C. Finally, hematoxylin was used to counterstain the nuclei. The Ki67 was quantitatively evaluated using the fluorescence microscope ( × 100 magnification).

### Statistical Analysis

Statistical analysis was performed using GraphPad Prism 6.0 software. Differences between groups were analyzed by Student’s t-test. Each assay was carried out in triplicate performed in parallel, and the results were expressed as mean ± standard deviation. *P*-values below 0.05 were considered significant.

## Results

### Characterization of FA-MOF/Buf NPs

According to previous reports (Akpinar and Yazaydin, 2017; Yang et al., 2018), a similar structured MOF (UiO-67) was synthesized *via* coordination between the metal ions (Zr^4+^) and the function group (-COOH) of 4,4′-biphenyldicarboxylic acid (H_2_BPDC). It is easy to get that the MOF NPs were formed based on the coordination effect between Zr^4+^ and the carboxyl group of 4,4′-DTBA. Through the 4,4′-DTBA, instead of H_2_BPDC, the MOF NP was endowed with redox-responsive properties. Meanwhile, the instability of the Zr–O bond allows it to show pH- responsive properties. Furthermore, it was then loaded with the drug (Buf) and underwent modification with FA-PEG. The general steps involved in the synthesis of FA-MOF/Buf are presented in Scheme 1.

TEM characterization was carried out for the MOFs to study their morphology. The images revealed that all samples exhibited uniform morphology. Furthermore, the morphology of MOF/Buf was similar to that of MOF, suggesting that the hydrophobic drug did not affect the intrinsic morphology of MOF (Supplementary Figure S1A). DLS was used to characterize the zeta potentials and hydrodynamic dimensions of MOFs. The results revealed that the size of FA-MOF/Buf was approximately 268 (±3.28) nm after FA-PEG modification (Table 1; Supplementary Figure S1B). The zeta potential of FA-MOF/Buf was characterized to be -11.67 (±1.38) mV (Table 1; Supplementary Figure S1C). The zeta potential of FA-MOF/Buf showed a negative state, probably due to the carboxylic ions of FA-PEG, demonstrating that successful modification with FA-PEG conferred FA-MOF/Buf a negative surface charge.

**TABLE 1:** Hydrodynamic (**‹D**_**h**_ **›**), polydispersity index (**μ**_**2**_ **/I** ^**2**^), and zeta potentials (*ξ*) of the MOF NPs

Sample	‹D_h_› (nm)^a^	PDI (μ_2_/I^2^)^a^	Zeta potential (*ξ*) (mV)^b^
MOF	179 (±1.98)	0.29	16.85 (± 0.78 )
MOF/Buf	210 (±2.78)	0.17	7.38 (±1.08)
FA-MOF/Buf	268 (±3.28)	0.26	−11.67 (±1.38)

UV–Vis characterization was carried out for Buf, FA-PEG, and FA-MOF/Buf (Supplementary Figure S3). Two absorption peaks at 278 and 335 nm were observed with FA-PEG. However, the absorption peak of FA-MOF/Buf demonstrated a slight red shift that appeared in the suspension when FA-MOF/Buf dissolved under ultrasonic vibrations. This is likely due to a few FA-PEG molecules entering into the tunnels and pores. Furthermore, the absorption peak of Buf was at 298 nm, but the absorption band of Buf was not seen in FA-MOF/Buf. This demonstrated that Buf was encapsulated in the MOF structure instead of adsorbing on the surface. The loaded Buf accounted for 17.4% of the weight of the NPs according to the UV–Vis results.

The N_2_ sorption isotherm after full activation in Supplementary Figure S4A shows that the MOF possesses a BET (Brunauer−Emmett−Teller) surface area of 862.16 m^2^/g. Such a big BET surface area not only proved its porosity but also showed that it could act as an ideal drug carrier for drug loading and delivery. Powder x-ray diffraction (PXRD) was used to characterize the crystallography of FA-MOF/Buf (Supplementary Figure S4B). The PXRD patterns of FA-MOF/Buf NPs corresponded well with that of the MOF, demonstrating the crystal integrity after MOF NP formation, Buf loading, and post-modification with the FA-PEG molecules. To further confirm the formation of FA-MOF/Buf NPs, FT-IR spectra of FA-MOF/Buf NPs and Buf were recorded (Supplementary Figure S4C). It is easy to understand that a new emerging peak around 1,112 cm^−1^ was witnessed at FA-MOF/Buf NPs which corresponded to the stretching of C–N of FA (Esfandyari-Manesh et al., 2016). Meanwhile, the peak centered at 1717 cm^−1^, which can be ascribed to the fact that C–O is stronger than original MOF (Esfandyari-Manesh et al., 2016). Those results proved that MOFs were successfully modified by FA.

### Redox and pH-Responsive Buf Release *in vitro*

The *in vitro* drug release was then explored to evaluate the redox-responsive Buf release ability from FA-MOF/Buf NPs. As shown in Supplementary Figure S2, it is obvious to see that the MOF NPs exhibit a GSH-dependent Buf release behavior. After exposure to C[GSH] = 10 mM, amounts of Buf released reached 62.8% at pH 7.4 and 78.8% at pH 6.5 at 24 h, which is 1.8-fold and 1.4-fold under the absence of GSH, respectively. To determine the release mechanism of MOF NPs, the Raman spectrum was used. In the Raman spectrum showed in Supplementary Figure S5A, two obvious peaks located at 500–560 cm^−1^ were seen at MOF NPs, which originate from the stretching vibration of S–S. (Hu et al., 1995; Su et al., 2007) However, the intensity of these two peaks became weak after being soaked in GSH solution. This phenomenon is due to unstable S–S bond (dissociation energy of 64.5 kcal/mol) (Kolano et al., 2004) being broken by the reductive GSH.

Then, the pH-responsive Buf release ability from FA-MOF/Buf NPs was also explored. As shown in Supplementary Figure S2, the NPs in PBS buffer solution (pH 5.5 + GSH 0 mM, pH 6.5+ GSH 0 mM and pH 7.4+ GSH 0 mM) revealed different Buf release behaviors. After exposure to pH 7.4+ GSH 0 mM, which represented the physiological conditions with neutral pH, only 34.2% of Buf encapsulated in MOF was released from NPs at 24 h, and only 39.7% of Buf was released from NPs at 48 h. However, when adding 10 mM GSH in PBS buffer, the Buf released 62.7% at 24 h and 73.1% (*p* < 0.05), respectively. In comparison, the amounts of Buf released reached 58.2% at pH 6.5+ GSH 0 mM at 24 h and 69.3% at 48 h, whereas, Buf released 78.7% at 24 h and 87.5% after adding 10 mM GSH in PBS buffer (*p* < 0.05). In addition to this, the Buf released 69.1% at 24 h and 81.2% at 48 h in pH 5.5+ GSH 0 mM buffer, indicating that an acidic condition and redox-responsiveness accelerates the dissociation of the MOF structure and facilitates Buf release. Since the tumor environment is weakly acidic, and GSH concentration is higher in cancer cells, it is conducive for Buf release from NPs and would enhance Buf anticancer efficacy. As mentioned in the Introduction section, the MOFs were assembled based on the coordination effect between metal ions and organic groups. In this research, the MOF NPs were based on the chemical bond between Zr^4+^ and -COOH of 4,4′-DTBA which was easily destroyed by protons. Hence, it could exhibit a pH-responsive performance. The PXRD was used to further explore the origin of pH-responsive performance. As depicted in Supplementary Figure S5B, the peaks of MOF NPs disappear after being stored at the acid environment, which may be cause by the Zr–O bond broken after the proton attack.

### *In vitro* Cytotoxicity

**FIGURE 1:**
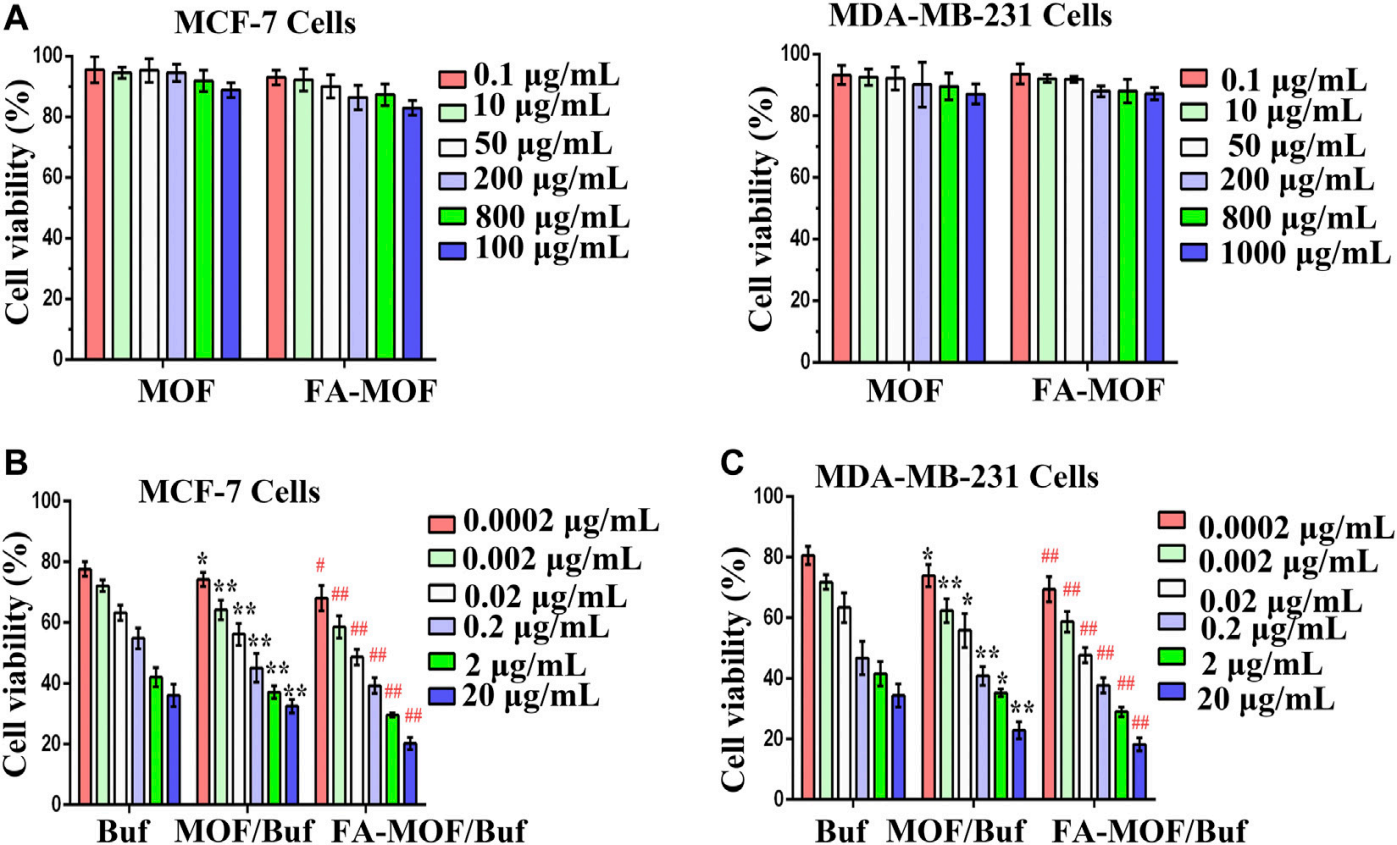
*In vitro* cytotoxicity of MOFs and FA-MOF against MDA-MB-231 and MCF-7 cells **(A)** Free Buf, Buf-labeled MOF (MOF/Buf), and Buf-labeled FA-MOF. (FA-MOF/Buf) against MCF-7 cells **(B)** and MDA-MB-231 cells **(C)** Concentrations used in the cytotoxicity experiments were based on the free Buf and its counterpart of loaded Buf into the MOF. Cell viability was measured by the CCK-8 assay by 48-h treatment. The data are presented as means ± SD (*n* = 3). *p < 0.05 and **p < 0.01 (MOF/Buf *vs.* Buf); ^#^ *p* < 0.05 and ^##^ *p* < 0.01(FA-MOF/Buf *vs.* MOF/Buf).

In order to assess the cytotoxicity of FA-MOF/Buf NPs, the CCK-8 assay was performed on MDA-MB-231 and MCF-7 human breast cancer cells. First, we examined the toxicity of MOF and FA-MOF at various concentrations on both cells and normal MCF-10A epithelial breast cells (Chen et al., 2017). As shown in Figure 1A; Supplementary Figure S6A, negligible toxicity was observed in these cells at 1,000 μg/ml, with cell viability > 90% at 48 h after treatment, indicating good biocompatibility of the NPs. For comparison, the cytotoxicity of free Buf, MOF/Buf and FA-MOF/Buf is also presented (Figures 1B,C). The same Buf concentrations were used for all samples. The cytotoxicity observed in cells was in the following order: Buf < MOF/Buf < FA-MOF/Buf. The results show that MOF/Buf and FA-MOF/Buf exhibit the same cytotoxicity on A549 cells and no statistical significance between them (Supplementary Figure S6B), indicating that Buf encapsulated in the MOF enhanced the cytotoxicity of Buf on cells. When Buf was loaded onto MOF NPs, its solubility was increased, and it was more easily absorbed by cells when in the form of NPs. Thus, an increased amount of Buf entered the cells, increasing the toxicity.

### Apoptosis Assay

**FIGURE 2:**
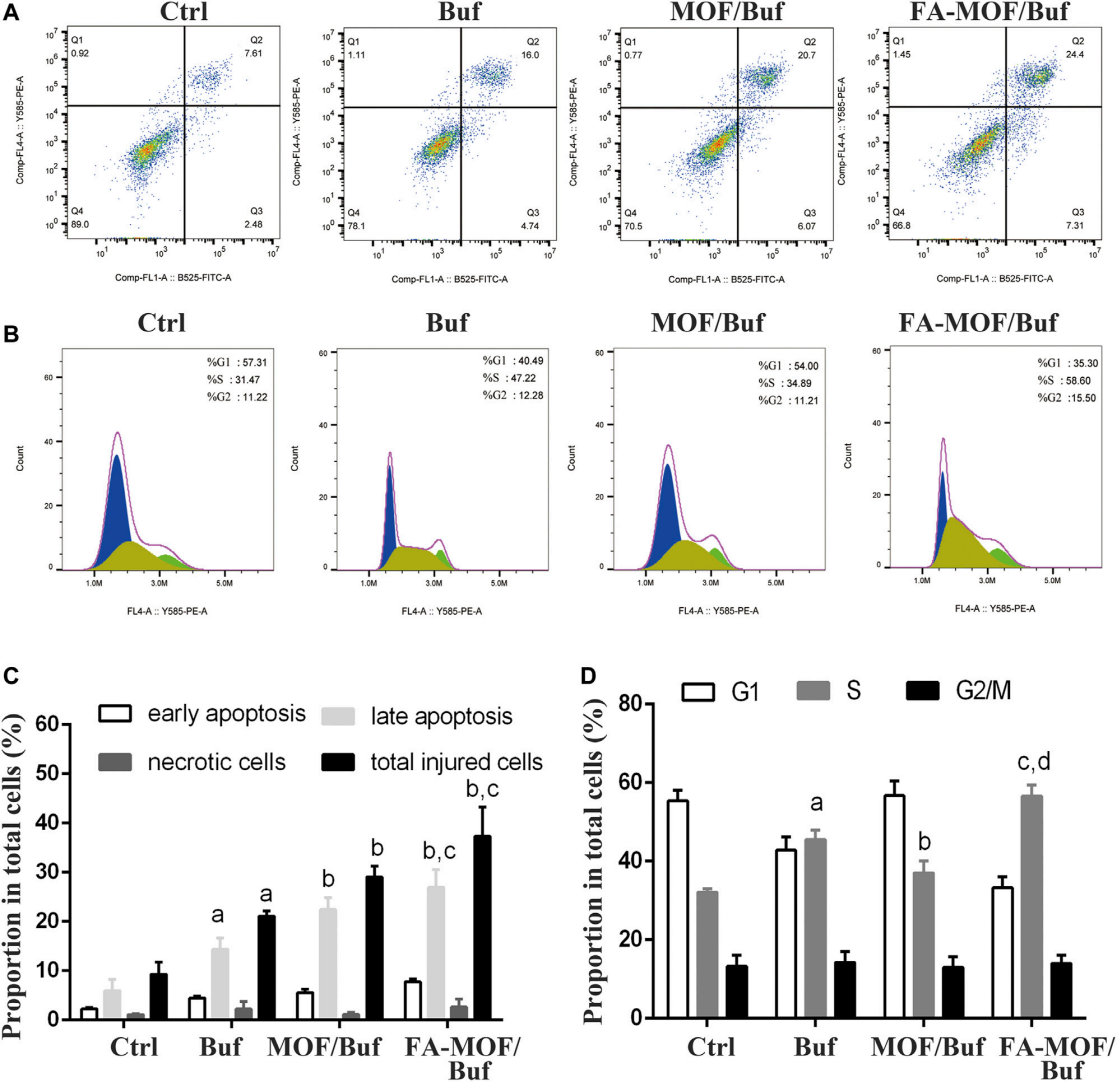
Cell apoptosis and cycle of MCF-7 cells after treatment with NPs. **(A)** Apoptosis analysis; **(B)** Cell cycle analysis; **(C)** Quantitative analysis of cell apoptosis, ^a^ *p* < 0.05 vs. ctrl, ^b^ *p*<0.01 vs. Buf, ^c^ *p*<0.05 vs. MOF/Buf (*n* = 3); **(D)** Quantitative analysis of cell cycle, ^a^ *p* < 0.01 vs. ctrl, ^b^ *p*<0.05 vs. Buf, ^c^ *p* <0.01 vs. Buf, ^d^ *p*<0.01.

The effects on MCF-7 cell apoptosis were quantified through flow cytometry analysis using the FITC annexin V/PI kit. As shown in Figures 2A,C, free Buf resulted in increased cell apoptosis compared with the control group, with an apoptosis rate of 16.0%. As expected, MOF/Buf and FA-MOF/Buf further increased apoptosis, with an apoptosis rate of 20.7 and 24.4%, respectively. The apoptosis rate of FA-MOF/Buf NPs was higher than that of free Buf mainly due to the increased cellular uptake through receptor-mediated endocytosis, as well as an increased intracellular concentration of Buf.

### Cell Cycle Analysis

The cell cycle distribution was quantified through flow cytometry analysis using the cell cycle and apoptosis kit. As shown in Figures 2B,D, most cells were distributed in the G1 phase in the control group. However, after treatment with free Buf, the proportion of cells in the S phase significantly increased from 31.47 to 47.22%, while the proportion of cells in the G1 phase decreased from 57.31 to 40.49%. As expected, after treatment with FA-MOF/Buf NPs, the proportion of cells in the S phase further increased to 58.6%, while the proportion of cells in the G1 phase decreased to 35.3%. This implies that the FA-MOF/Buf NPs were more effective in inducing cell cycle arrest compared with free Buf.

### *In vitro* Cellular Uptake

**FIGURE 3:**
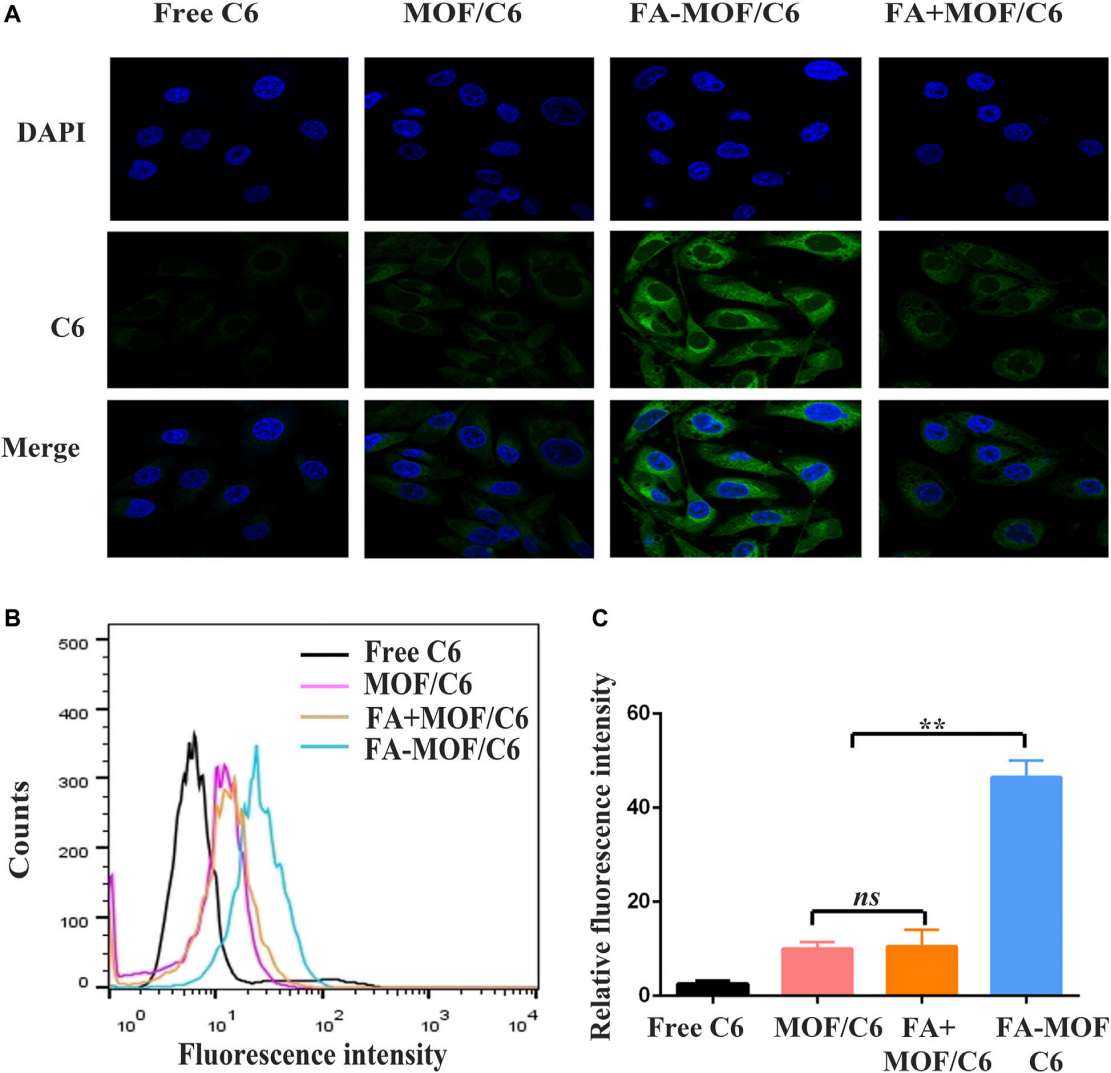
*In vitro* cellular uptake and folic acid competition assay. MCF-7 cells incubation with free C6, C6-labeled MOF (MOF/C6), C6-labeled FA-MOF (FA-. MOF/C6), and C6-labeled MOF combined with 1 mM folic acid (FA + MOF/C6) for 2 h. **(A)** Fluorescence images of cells were obtained by using a confocal laser scanning microscope; the scale bar was 20 µm. **(B)** Uptake efficiency on MCF-7 cells was obtained by flow cytometry and quantitative analysis. **(C)** **p < 0.01.

The ability of the drug delivery system to target tumor cells is an essential function of tumor-targeting NPs. As described earlier, the FR receptor is overexpressed in many cancer cells, which is partly ascribed to the targeting moieties for MCF-7 cells. In this study, cellular uptake of NPs was observed by replacing Buf with C6 and was evaluated in MCF-7 cells by confocal laser scanning microscopy and flow cytometry. As shown in Figure 3Athe uptake of FA-MOF/C6 was significantly higher compared with other treatments. These could have resulted from the FA-targeting FRs, which increased the cellar uptake of NPs. In addition, flow cytometry was also used to evaluate the cellular uptake ability. As shown in Figures 3B,C, the FA-MOF/C6 can effectively enter cells as revealed by the strong fluorescence compared with the control group (free C6). However, the fluorescence intensity in FA + MOF/C6 exhibited little difference compared with MOF/C6, mainly due to folic acid competition and blocking targeting ability. These results indicate that FA-MOF could improve cell uptake ability and enhance the cancer cell targeting ability of the drug, most likely due to the high expression of the FR receptor in MCF-7 cells.

### *In vivo* Biodistribution Examination

**FIGURE 4:**
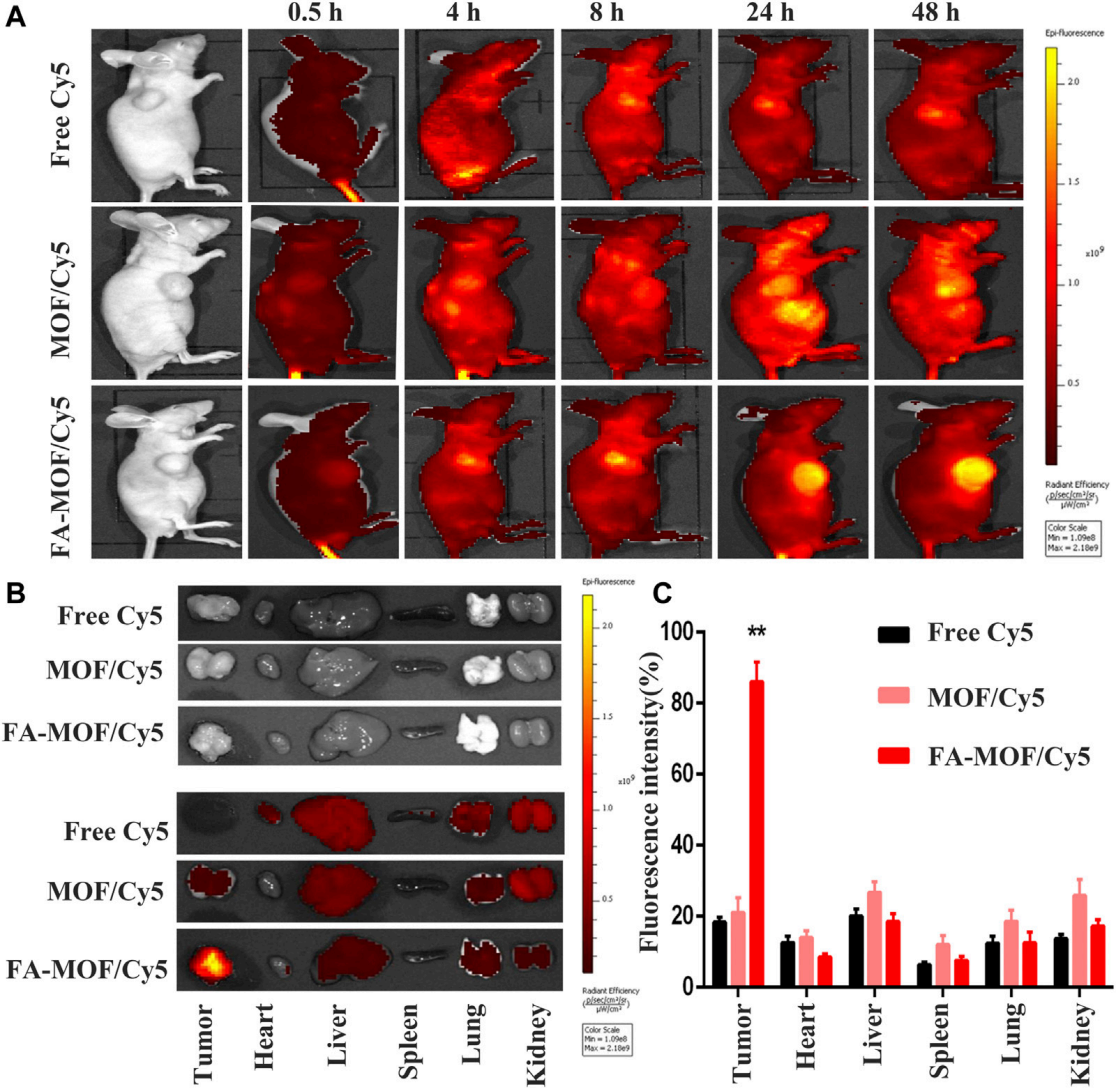
*In vivo* fluorescence imaging of tumor-bearing mice at 0.5, 4, 8, 24, and 48 h after intravenous injection; the decrease of fluorescent intensities was indicated by the color bar from yellow to red **(A)**. Main organs, including heart, liver, spleen, lungs, and kidneys, and tumors were collected from mice at 48 h post injection for fluorescence images **(B)**. Biodistributions of Cy5, MOF/Cy5, and FA-MOF/Cy5 in tumor issues and different organs. **(C)** **p < 0.01.

The whole-body fluorescence images of mice are shown in Figure 4As described previously, the FA-modified NPs enhanced the internalization of drugs into tumor cells. The tumor-targeting capability was evaluated using Cy5-encapsulated targeting FA-MOF/Cy5 NPs and non-targeting MOF/Cy5 NPs, which were intravenously injected into 4T1 tumor–bearing mice through the tail vein. The results demonstrated the tumor accumulation of FA-MOF/Cy5 and MOF/Cy5 at various time intervals. At 0.5 h post injection, the fluorescence signals were observed throughout the body in both groups. At 4 h post injection, the fluorescence intensity was augmented in the tumor site. With time, the fluorescence intensity of the FA-MOF/Cy5 group at the tumor area was stronger than that of the MOF/Cy5 group. As expected, the fluorescence signals of the FA-MOF/Cy5 group showed a longer retention time and were retained in the tumor even at 48 h post injection (Figure 4A). On the contrary, the fluorescence signals only accumulated in the tumor tissue in the MOF/Cy5 group at 48 h post injection, with most of the accumulated fluorescence localized in the liver and kidneys. This may be due to some free drugs released and cleaved from NPs in the circulation period. Additionally, the fluorescence intensity in the tumor tissue was much stronger in the FA-MOF/Cy5 group than in other organs, presumably due to FA modification (Figures 4B,C). Our results indicated that the tumor-targeting properties and accumulation of FA-MOF/Cy5 could be attributed to the EPR effect and FA modification.

### *In vivo* Antitumor Efficacy

**FIGURE 5:**
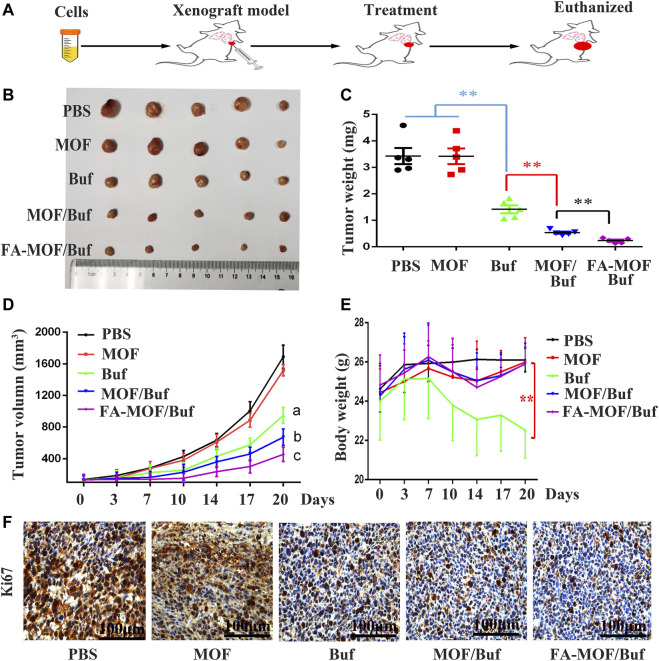
Anticancer efficacy on the subcutaneous tumor mouse model. **(A)** Flow chart of the experiment. **(B)** Representative tumor tissues excised from mice at the end of treatment. **(C)** Tumor weight at the end of treatment. **(D)** Body weight of tumor-bearing mice during the period of treatment. **(E)** Tumor volume with time after cancer cell inoculation. **(F)** Expression of Ki67 in cancer tissues, measured by IHC staining (scale bar: 100 μm). Data are expressed as mean ± SD (*n* = 6), **p < 0.01, ^a^ *p* < 0.01 Buf vs. PBS, ^*b*^ *p* < 0.05 MOF/Buf vs. Buf, ^c^ *p <* 0.05 FA-MOF/Buf vs. MOF/Buf.

**FIGURE 6:**
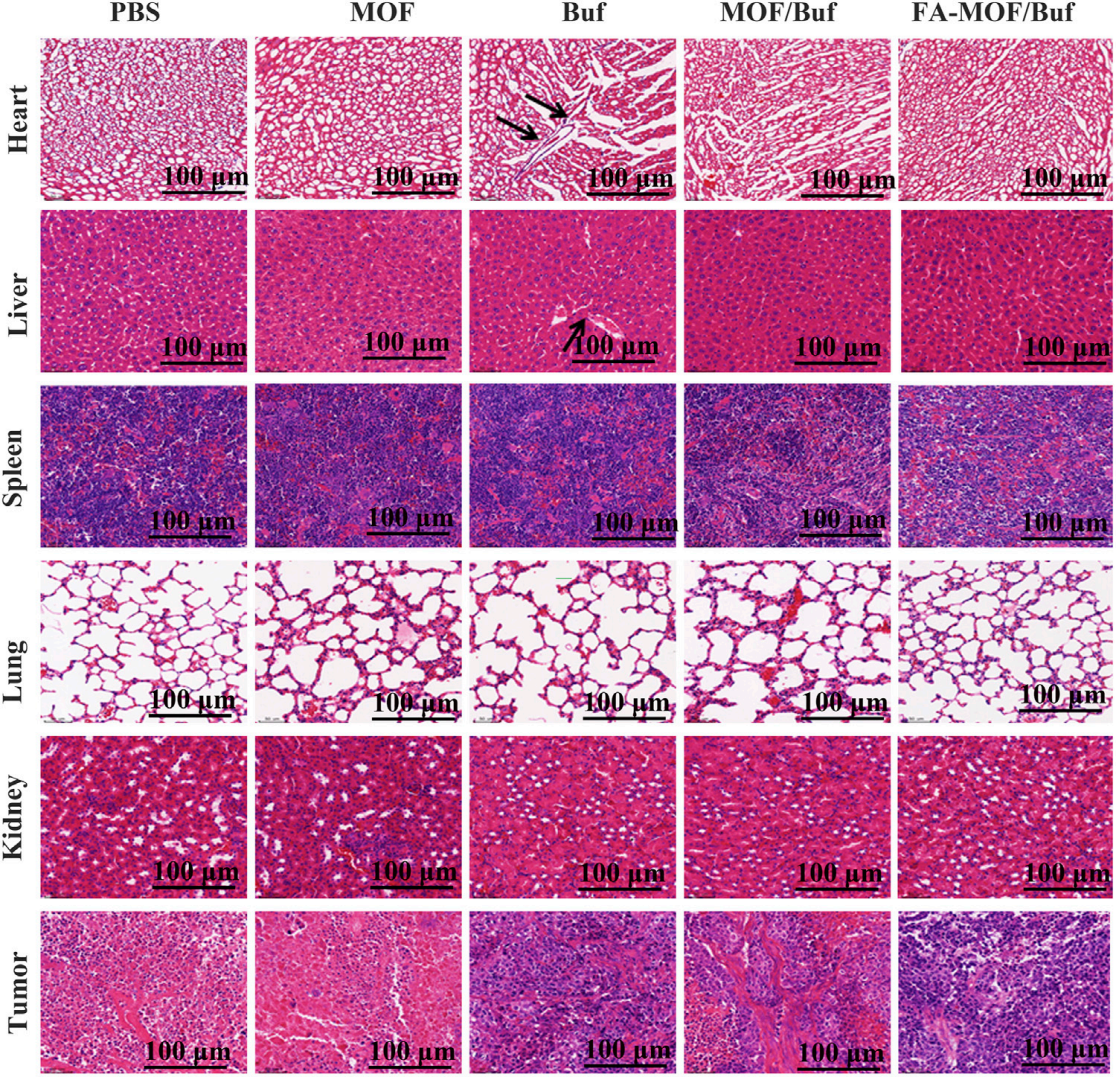
Histopathological examination of major organs (heart, liver, spleen, lungs, and kidneys) and tumor tissues after treatment on the xenografted mice (scale bar: 100 μm).

Buf-loaded FA-modified MOF NPs (FA-MOF/Buf) showed a significantly improved antitumor efficacy in the 4T1 tumor model. As shown in Figure 6, empty carrier MOFs did not show an antitumor effect compared with the PBS control group. However, after loading Buf (2 mg/kg), the tumor growth was significantly inhibited compared with that of the control group. Moreover, all the Buf-loaded NPs presented a greater efficacy on tumor growth suppression compared with Buf alone (Figures 5B,C) The tumor volume was prominently reduced (Figure 5D), which may be due to the accumulation of bigger NPs in the tumor tissue, rather than small-molecule drugs based on EPR effects (Cao et al., 2016). Interestingly, the same phenomenon was observed in the *in vitro* cell cytotoxicity. Both results demonstrated improved antitumor efficacy. In addition, the FA-modified group (FA-MOF/Buf) demonstrated the greatest inhibition of tumor growth, mainly due to the tumor-targeting capability of NPs functionalized by FA and the macromolecular-based EPR effects. Moreover, the weights of all mice were measured after treatment. There were no remarkable changes in the PBS control group, MOF group, MOF/Buf group, and FA-MOF/Buf group, whereas the weight was reduced in the free Buf group after undergoing treatment twice, likely due to the toxicity of Buf (Figure 5E). The Ki67 expression in tumor tissues was measured by IHC, and the results show that the free Buf, MOF/Buf, and FA-MOF/Buf groups had less positive staining compared to MOF and PBS control groups. Moreover, the Ki67 expression in the FA-MOF/Buf group was minimal, followed by MOF/Buf group, and then free Buf group (Figure 5F). These results indicated that the efficacy of antitumor was FA-MOF/Buf  > MOF/Buf > MOF and PBS.

Finally, we analyzed histopathological changes in major organs (heart, liver, spleen, lungs, and kidneys) and tumor tissues used H&E staining. Results indicated that there are some changes in the heart and liver of the free Buf group. Cardiomyocyte necrosis, connective tissue proliferation, and liver cell necrosis, among other changes, were observed. However, in the control group and NP group, no apparent damages in major organs were appreciated. In addition, necrotic and apoptosis areas in the tumor tissue were much larger in the treatment groups than in the control groups, with the FA-MOF/Buf group demonstrating the most obvious changes (Figure 6). Moreover, the Ki67 expression in major organs (liver, lungs, and kidneys) was evaluated; the results showed no significant difference in all groups, indicating the safety of MOF materials (Supplementary Figure S7).

## Discussion

Buf is a natural product with digoxin-like immune activity and has antitumor effects by inducing apoptosis and inhibiting cell proliferation (Li et al., 2011). However, it is not widely used due to its poor solubility, high toxicity, fast metabolism, and short half-life (Hu et al., 2014). Nanodrug delivery systems effectively improve efficacy, decrease toxicity, and alleviate side effects through sustained drug release at the tumor site.

Nanodrug delivery systems are used to enhance permeability and retention through passive or active targeting mechanisms for delivering drugs to the tumor site (Choi et al., 2017). The tumors are rich in blood vessels that provide nutrients for growth. At the same time, tumor blood vessels have poor structural integrity and wider gaps between walls, which allow NPs to efficiently enter and accumulate at the tumor site (Yu et al., 2016). Nanoscale metal-organic frameworks (nMOFs), with molecular modularity, structural tunability, intrinsic porosity, tunable stability, and biocompatibility, are ideally suited for biomedical applications, particularly cancer treatments (Ni et al., 2020). The type of NP used in our study is a pH-sensitive and redox-responsive MOF NP which had been modified with FA to promote active targeting of FRs on the tumor surfaces. This modification could improve drug accumulation and release in the tumor site. The FA-MOF/Buf displayed higher reduction in tumor growth compared with the free Buf. Modification with antibodies or other targeting molecules on the NP surface can also help deliver drugs precisely to the tumor site and increase the local drug concentration (Tang et al., 2018).

In this study, we successfully prepared a multifunctional drug delivery system, FA-MOF/Buf NPs, which allows for specific and targeted identification of breast cancer through a one-pot process. The obtained NPs exhibited high drug loading capacity, low cytotoxicity, uniform size, and high stability under physiological conditions to protect the encapsulated Buf from loss before its drug delivery to cancer tissues *via* the EPR effect. Additionally, the pH-sensitive release can accelerate Buf release from NPs in the low pH environment of cancer tissues. Moreover, the FA-modified NPs showed higher intracellular uptake and enhanced cytotoxicity against breast cancer cells *in vitro*. The *in vivo* fluorescence imaging results revealed that the FA-MOF/Buf exhibited excellent drug accumulation in tumor regions, suggesting good targeted anticancer effects. In the tumor-bearing nude mice model, the FA-MOF/Buf NPs demonstrated significantly improved antitumor activity and minor toxicity and side effects on normal organs, demonstrating their excellent biocompatibility and safety compared with the direct administration of free Buf. Therefore, our developed Buf-loaded, pH-sensitive, tumor-targeting drug delivery system, FA-MOF/Buf, is a promising platform for breast cancer therapy.

## Conclusion

In this study, we designed a pH-sensitive and redox-responsive MOF drug carrier. We then incorporated Buf into the MOF and added an FA modification to the MOF surface. The obtained FA-MOF/Buf NPs quickly degraded in low-pH environments of tumors and demonstrated high intracellular uptake and enhanced cytotoxicity against breast cancer cells *in vitro*. FA-MOF/Buf exhibited excellent drug accumulation in the tumor regions, allowing targeted anticancer effects and improved antitumor activity *in vivo*.

## Data Availability

The original contributions presented in the study are included in the article/Supplementary Material, further inquiries can be directed to the corresponding authors.
