# Supplementary material for: Folic Acid–Functionalized Metal-Organic Framework Nanoparticles as Drug Carriers Improved Bufalin Antitumor Activity Against Breast Cancer
Source: Front Pharmacol. 2022 Jan 18;12:747992. doi: 10.3389/fphar.2021.747992 (PMC8805731; doi:10.3389/fphar.2021.747992)
Supplement: Supplementary file 1 [file DataSheet1.docx]

Supplementary Material

*for*

**Folic acid-functionalized metal-organic framework nanoparticles as drug carriers improved bufalin antitumor activity against breast cancer**

**Hairong Zeng^1,3†^, Chao Xia^2†^, Bei Zhao^3†^, Mengmeng Zhu^2^, HaoYue Zhang^5^, Die Zhang^1^, Xin Rui^4^*, Huili Li^2^*, Yi Yuan^1^***

^1^ *Department of Pharmacy, Putuo Hospital, Shanghai University of Traditional Chinese Medicine, Shanghai 200062, China;*

*^2^ Engineering Research Center for Nanophotonics & Advanced Instrument, Ministry of Education, School of Physics and Electronic Science, East China Normal University, Shanghai, 200241, China;*

*^3^ Institute of Interdisciplinary Integrative Medicine Research, Shanghai University of Traditional Chinese Medicine, Shanghai, 201203, China;*

*^4^Baoshan Branch, Shuguang Hospital Affiliated to Shanghai University of Traditional Chinese Medicine, Shanghai, 201900, China;*

*^5^* *Colorectal Disease Center of Nanjing Hospital of Chinese Medicine Affiliated to Nanjing University of Chinese Medicine, Nanjing 210022, Jiangsu Province, China;*

**^†^ *These authors have contributed equally to this work and share first authorship.***

* **Correspondence:**
* Corresponding author: Yi Yuan, [yuanyi0625@163.com](mailto:yuanyi0625@163.com);

Huili Li, [hlli@phy.ecnu.edu.cn](mailto:hlli@phy.ecnu.edu.cn);

Xin Rui, riyusing@163.com.

**This file contains 7 supplementary figures.**

**Supplementary Figure 1**


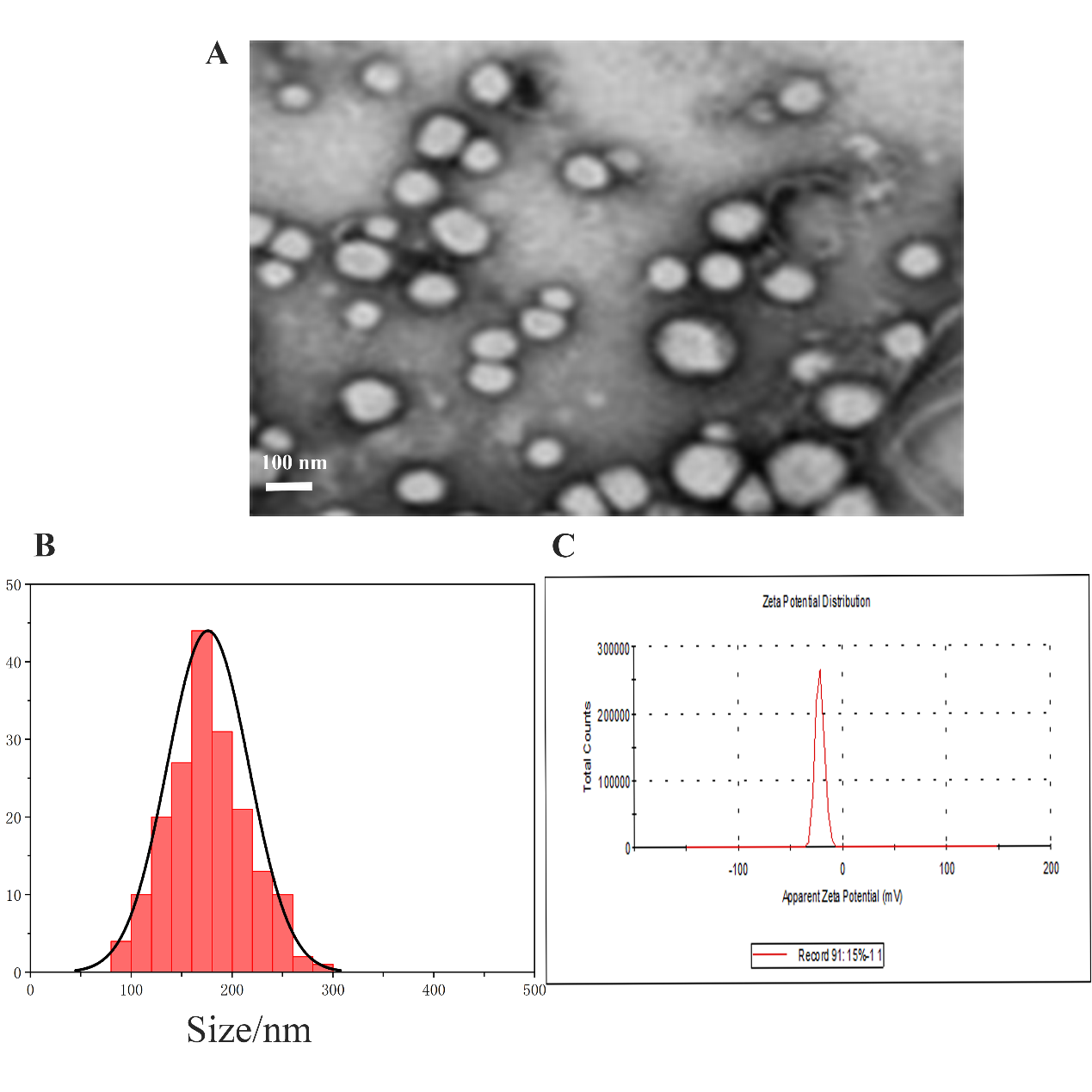


**Supplementary Figure 1**. Characteristics of FA-MOF/Buf NPs. (A) TEM images of NPs, (B) Size distribution of NPs, (C) Zeta potential of NPs.

**Supplementary Figure 2**


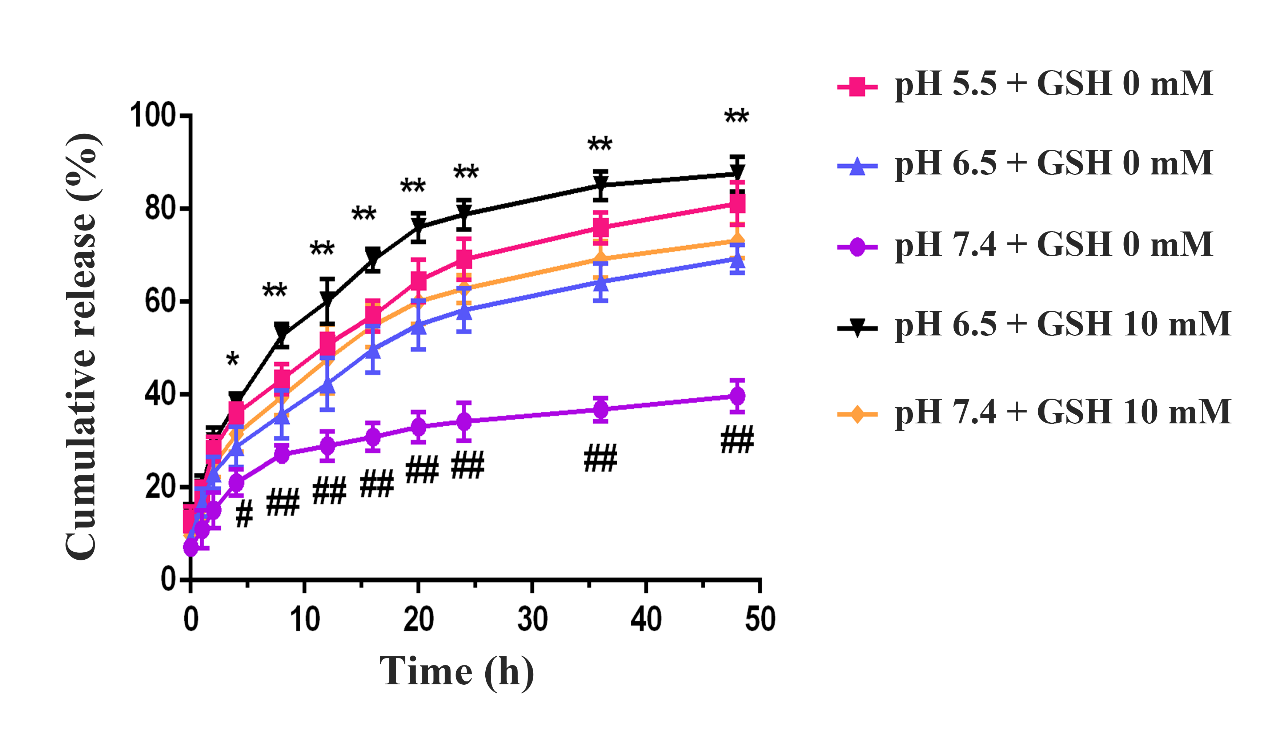


**Supplementary Figure 2.** In vitro Buf release curve from FA-MOF/Buf NPs in different pH with or without GSH 10 mM at 37 °C. Date presented as mean ± SD of three parallel experiments. **P﹤*0.05, and ***P﹤*0.01 (pH 6.5+ GSH 0 mM vs. pH 6.5+ GSH 10 mM); ^#^*P﹤*0.05, and ^##^*P﹤*0.01(pH 7.4+ GSH 0 mM vs. pH 7.4+ GSH 10 mM).

**Supplementary Figure 3**


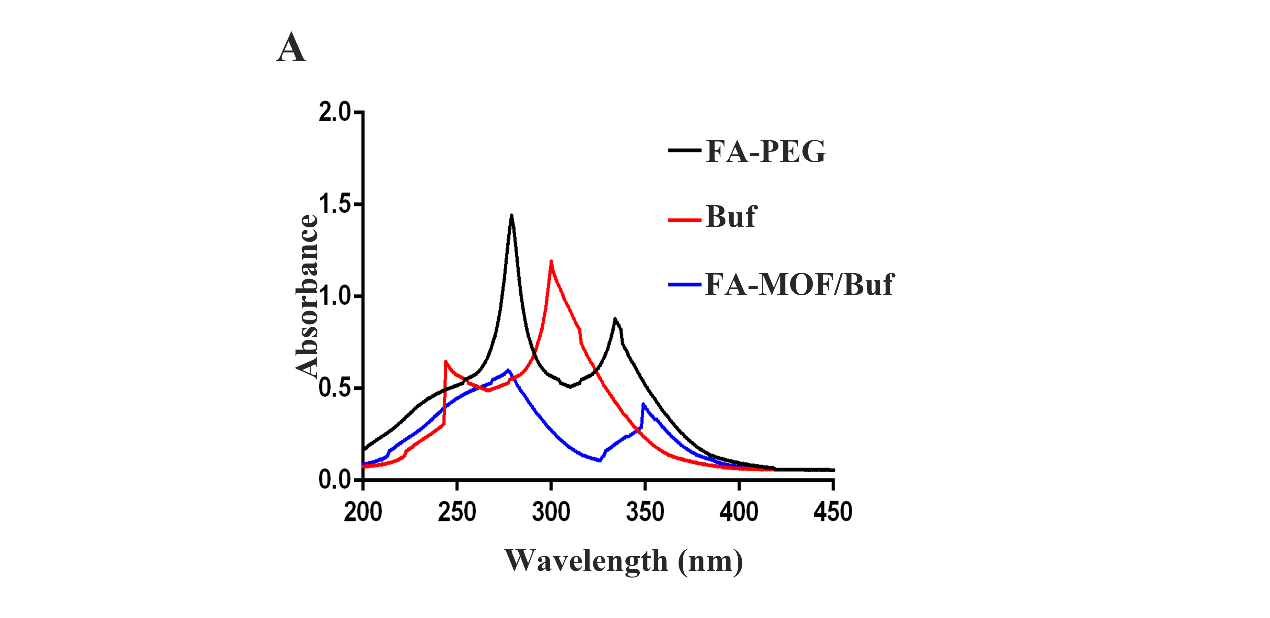


**Supplementary Figure 3**. UV-Vis spectra of FA-PEG, Buf and FA-MOF/Buf.

**Supplementary Figure 4**


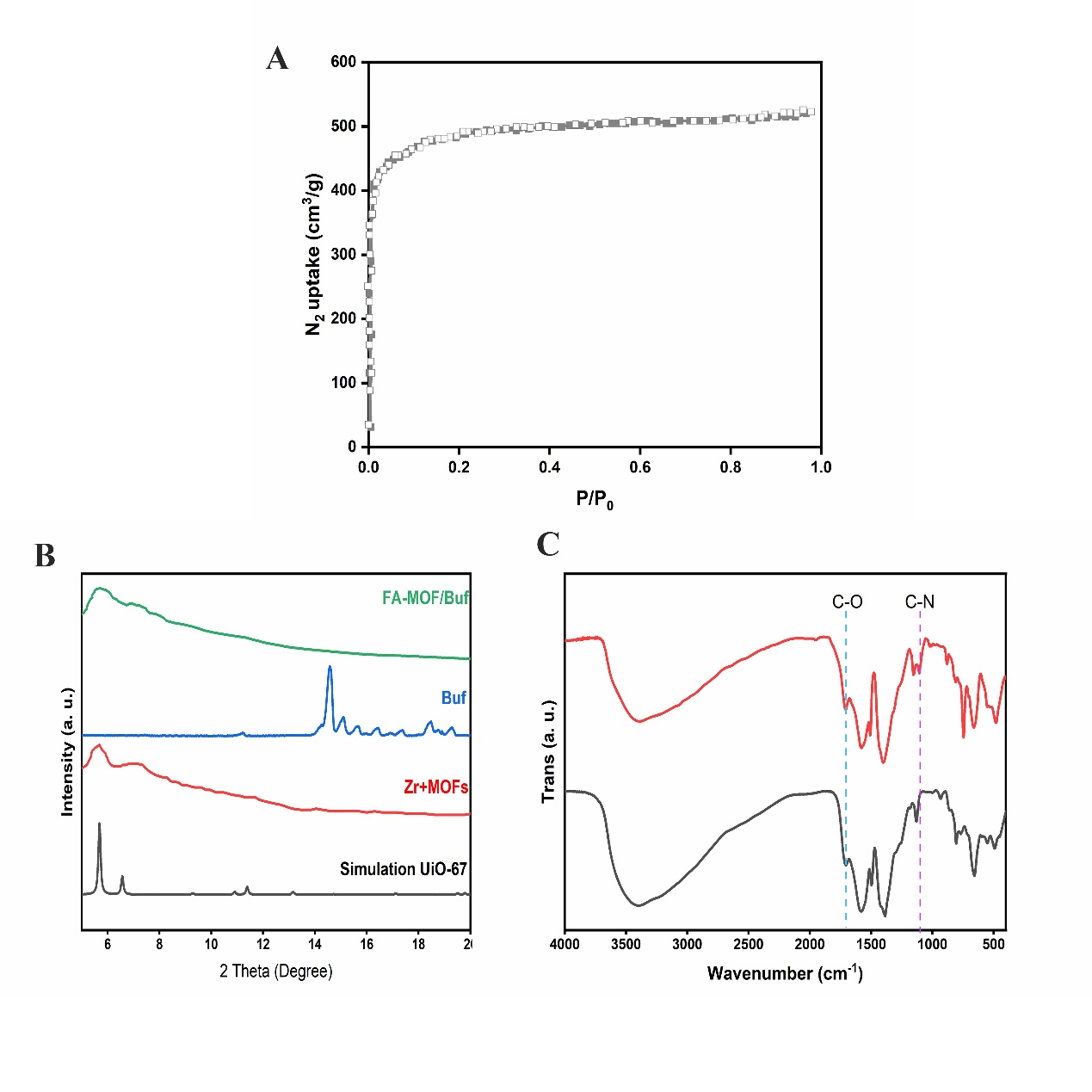


**Supplementary Figure 4.** (A) Nitrogen adsorption isotherms of MOFs. (B) The PXRD results of MOFs (red), Buf (blue) and FA-MOF/Buf (green). (C) The FT-IR of MOFs (black) and FA-MOF/Buf (red).

**Supplementary Figure 5**


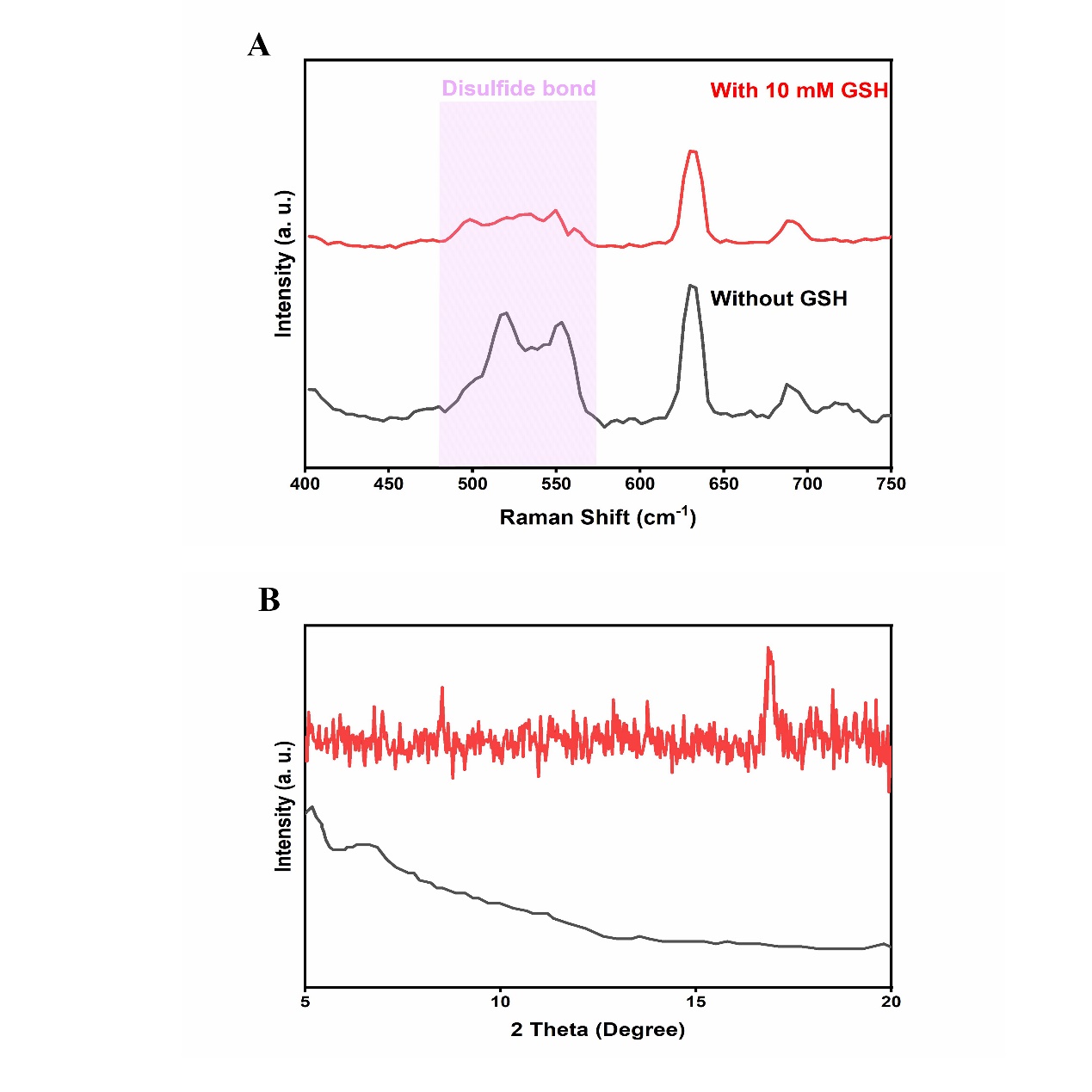


**Supplementary Figure 5. (A)** Raman spectrum of MOFs without GSH (black) and with 10 Mm GSH (red). (B) The PXRD of MOFs after storage in different PBS (black, pH=7.4, red, pH=5.5) for 24h.

**Supplementary Figure 6**


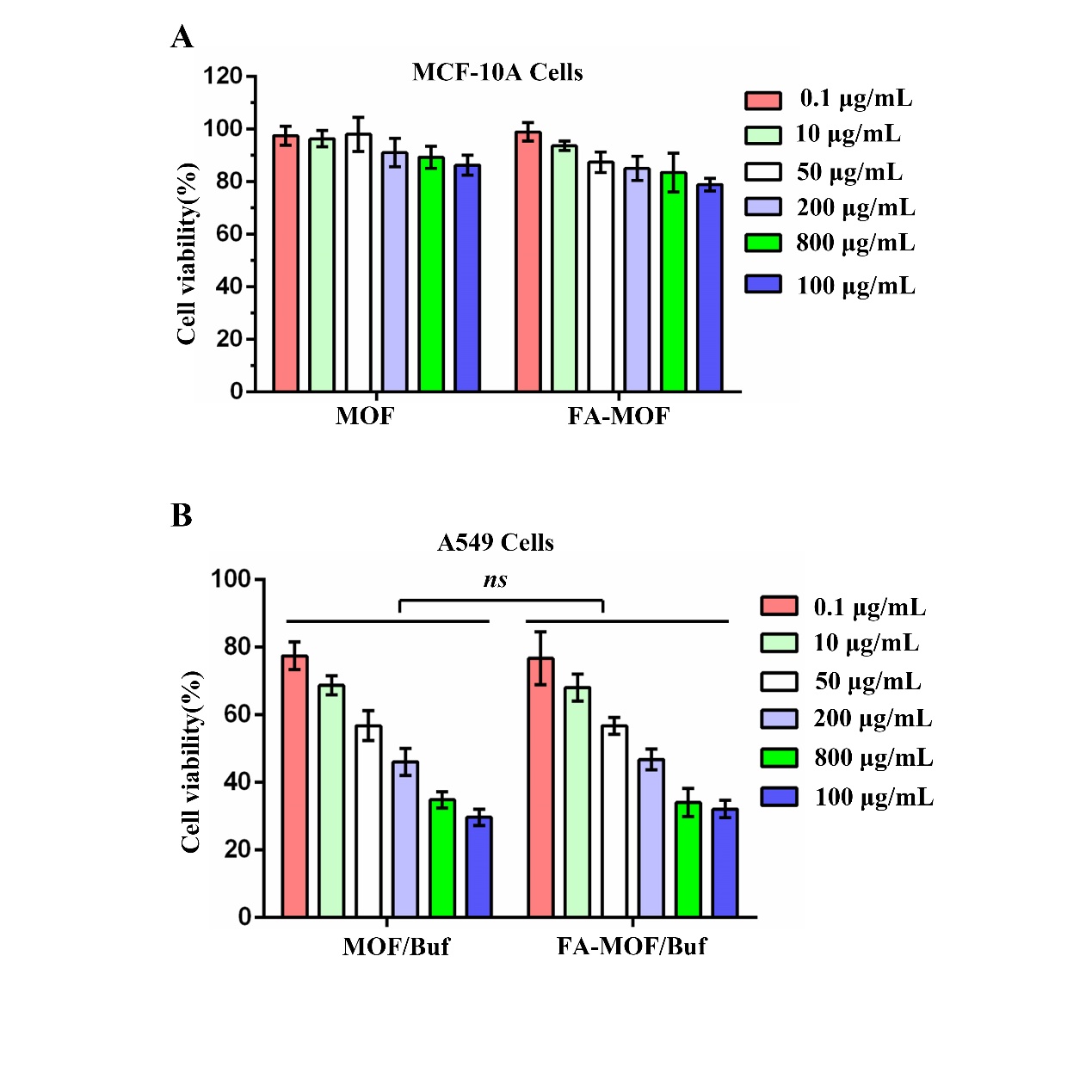


**Supplementary Figure 6.** In vitro cytotoxicity. (A) MOF and FA-MOF on normal MCF-10A epithelial breast cells. (B) In vitro cytotoxicity of MOF/Buf and FA-MOF/Buf on A549 cells (folate receptor-negative A549 lung carcinoma cells). Cell viability was measured by CCK-8 assay by 48 h treatment. The date presented as means ±SD (n=3).

**Supplementary Figure 7**


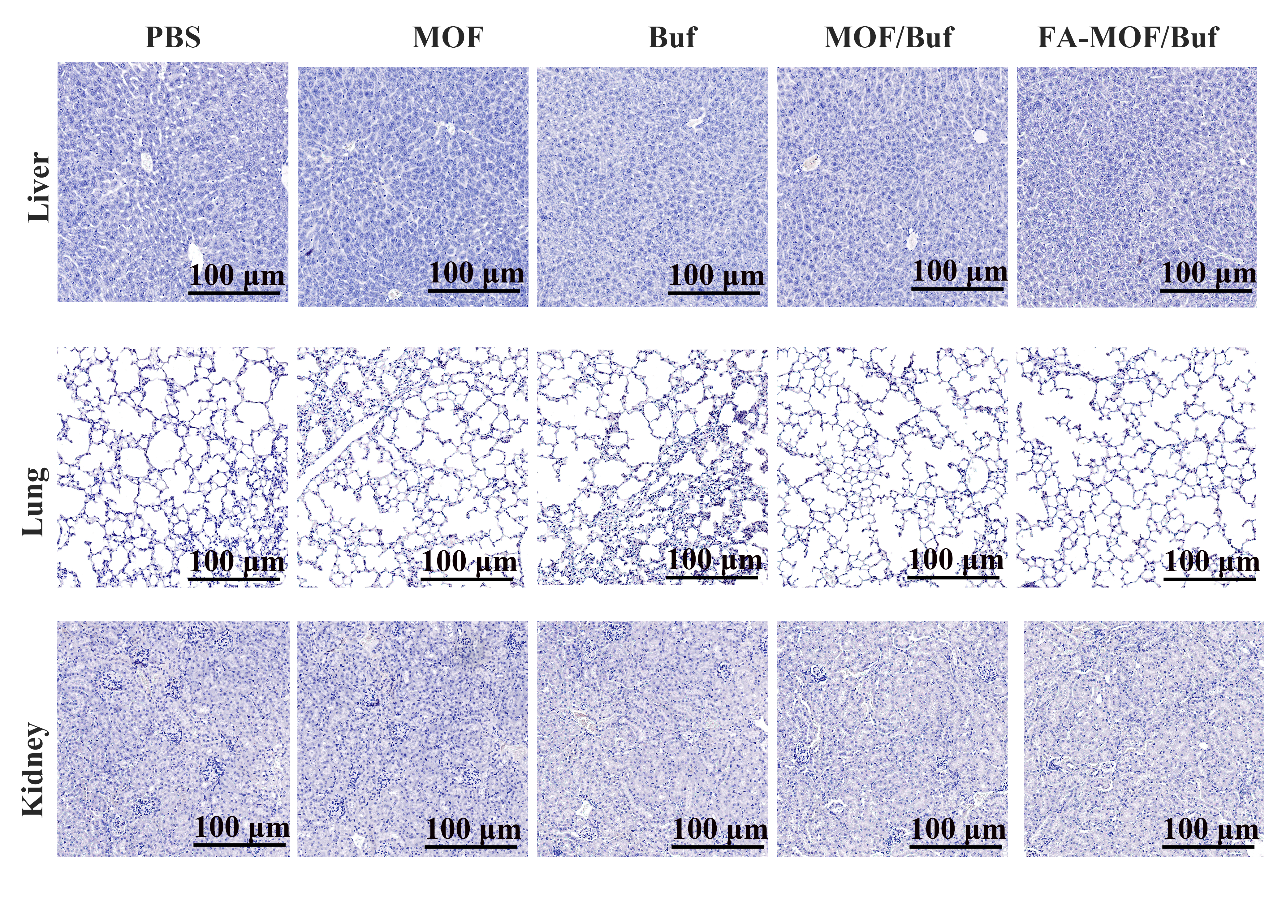


**Supplementary Figure 7.** The Ki67 expression in major organs (liver, lung, and kidney) was measured by IHC (scale bar: 100 μm).
